# A new Approach to Identify Gene-Environment Interactions and Reveal New Biological Insight in Complex traits

**DOI:** 10.21203/rs.3.rs-3338723/v1

**Published:** 2023-10-05

**Authors:** Xiaofeng Zhu, Yihe Yang, Noah Lorincz-Comi, Gen Li, Amy Bentley, Paul S de Vries, Michael Brown, Alanna C Morrison, Charles Rotimi, W. James Gauderman, DC Rao, Hugues Aschard

**Affiliations:** 1 Department of Population and Quantitative Health Sciences, Case Western Reserve University, Cleveland, Ohio, USA; 2 Center for Research on Genomics and Global Health, National Human Genome Research Institute, National Institutes of Health, Bethesda, Maryland, USA; 3 Human Genetics Center, Department of Epidemiology, Human Genetics, and Environmental Sciences, School of Public Health, The University of Texas Health Science Center at Houston, Houston, Texas, USA; 4 Biostatistics, Department of Preventive Medicine, University of Southern California, Los Angeles, CA, USA; 5 Division of Biostatistics, Washington University School of Medicine, St. Louis, MO, USA; 6 Institut Pasteur, Université Paris Cité, Department of Computational Biology, F-75015 Paris, France

## Abstract

There is a long-standing debate about the magnitude of the contribution of gene-environment interactions to phenotypic variations of complex traits owing to the low statistical power and few reported interactions to date. To address this issue, the CHARGE Gene-Lifestyle Interactions Working Group has been spearheading efforts to investigate G×E in large and diverse samples through meta-analysis. Here, we present a powerful new approach to screen for interactions across the genome, an approach that shares substantial similarity to the Mendelian randomization framework. We identified and confirmed 5 loci (6 independent signals) interacting with either cigarette smoking or alcohol consumption for serum lipids, and empirically demonstrated that interaction and mediation are the major contributors to genetic effect size heterogeneity across populations. The estimated lower bound of the interaction and environmentally mediated contribution ranges from 1.76% to 14.05% of SNP heritability of serum lipids in Cross-Population data. Our study improves the understanding of the genetic architecture and environmental contributions to complex traits.

Current genome-wide association studies (GWAS) focus on detecting genetic variants that lead to different phenotype means across genotype groups ^1,2^, and have identified a large number of genetic loci that, in some cases, explain large proportions of the trait’s SNP-heritability ^3–5^. While it is commonly agreed that complex traits are influenced by genetic and environmental factors and their interactions ^6–9^, there is a long-standing disagreement about the magnitude of the G×E contribution to heritability because of different theoretical models and assumptions^10,11^. As pointed out in ^12^, arbitrarily defined parameterizations of genetic effects with non-additive gene actions may explain the same degree of genetic variation as the currently prevailing additive model. Thus, while using additive genetic models such as polygenic risk scores to predict individual quantitative or qualitative phenotypes has become standard ^5^, these models may not be fully informative in understanding genetic architecture.

Interactions are often studied secondarily in comparison to additive variance, whose advantage is to explain most of the correlations among relatives and fit natural selection models well ^10,13^. Theoretical studies have demonstrated that a significant portion of variance can be explained by an additive model even when the genetic contribution to a phenotype is purely through G×E ^14^. This limitation is one of the key factors explaining the low power of approaches modeling interactions conditional on additive variance. As a result, studies focusing on detecting G×E at the genome-wide level are seldom considered as primary analyses. Instead, the joint evidence of main genetic and G×E effects, in addition to the G×E alone, is tested in the Gene-Lifestyle Interactions (GLI) Working Group within the Cohorts for Heart and Aging Research in Genetic Epidemiology Consortium (CHARGE)^9,15^, where only a modest number of genetic loci have been identified through testing for G×E alone ^16–19^. The joint test limits our ability to determine to what degree the currently identified loci reflect evidence for G×E contribution, making it difficult to understand the precise interplay between genetic and environmental factors.

Concurrently, Mendelian Randomization (MR) has been developed and widely applied to study causal relationships between risk exposures and outcomes in the post-GWAS era ^20,21^. Although MR approaches have been used to explain G×E ^22^ and assess risk factor interactions^23^, the underlying connection between testing pleiotropic variants in the MR framework and the detection of G×E is currently unclear. Here, we conceptually connect G×E with the MR framework, illuminate their similarities and demonstrate that the test of horizontal pleiotropy in MR ^24,25^ can be used for detecting G×E. Based on this principle, one can identify novel G×E using existing summary statistics without needing costly and time-consuming new analyses from all cohorts. We applied this approach to the summary statistics from the Global Lipids Genetics Consortium study (GLGC, n=1.65M) ^3^ and the summary statistics in the interaction analysis with cigarette smoking and alcohol drinking in the CHARGE GLI working group ^17^, with replication using direct interaction tests performed in the UK Biobank (UKBB) data. Although the UKBB data accounted for about one third of sample in the GLGC consortium, theoretical work suggests that such replication is statistically independent (Supplementary Note).

## Results

### Testing G×E and mediation based on Mendelian Randomization (MR)

Traditionally a genome wide interaction study (GWIS) with an environmental exposure on a quantitative trait *Y* is modeled through a linear regression:

$$Y=\beta_{0}+\beta_{1}\text{G}+\beta_{2}\text{E}+\beta_{3}\text{G}\times \text{E + }\epsilon ,$$

where $\beta_{1}$, $\beta_{2}$ and $\beta_{3}$ correspond to the ‘main’ effect of G (in the presence of E), the main effect of E and the interaction effect of G×E, respectively, and ϵ is a random noise. Here G, E, and G×E represent a genotype value, environmental factor, and their interaction, respectively. For simplicity, we do not include any covariates, but this does not affect the general conclusion. The interaction effect is evaluated by the *direct test* statistic $T_{direct}={\hat{\beta }}_{3}^{2}/\text{var}\left({\hat{\beta }}_{3}\right)$, where ${\hat{\beta }}_{3}$ refers to the estimate from the regression model (1). Theoretical work indicates that the test statistics for the main effect $\beta_{1}=0$ and the interaction effect $\beta_{3}=0$ are correlated, with the correlation coefficient equal to $-\mu_{E}/\sqrt{\mu_{E}^{2}+\sigma_{E}^{2}}$, where $\mu_{E}$ and $\sigma_{E}^{2}$ are the mean and variance of the environmental factor in the data ^14^. However, the power of the direct test is usually low because of the collinearity between G and G×E which induces a covariance between the estimates of $\beta_{1}$ and $\beta_{3}$. This covariance produces uncertainty (i.e. larger standard error) which by itself reduces power for testing either $\beta_{1}$ or $\beta_{3}$, even if the underlying true model includes G×E alone (i.e., $\beta_{1}=0$ and $\beta_{3}\neq 0$) ^10,14^.

In practice, a GWAS is routinely conducted first when studying the genetic contribution to a trait, which is typically done through a linear regression model without including environmental factors, i.e.,

$$Y=\alpha_{0}+\alpha \text{G}+\epsilon ,$$

where we refer to α as the ‘marginal’ effect from a GWAS (in the absence of E) to differentiate it from the main effect $\beta_{1}$ in model (1). We show that $\alpha -\beta_{1}=\frac{\rho \sigma_{E_{1}}}{\sigma_{G_{1}}}\beta_{2}+\left(\mu_{E_{1}}+\frac{\rho \sigma_{E_{1}}}{\sigma_{G_{1}}}\right)\beta_{3}$, where ρ is the mediation contribution of G through E, $\mu_{E_{1}}$, $\sigma_{E_{1}}$, and $\sigma_{G_{1}}$ represent the environmental mean, standard deviation, and genotype standard deviation in GWAS data, respectively, suggesting that testing the hypothesis ${\text{H}}_{0}:\alpha -\beta_{1}=\;0$ for the difference between the marginal and main effects is equivalent to testing for the combined effect of *G* × *E* and mediation, and further reduces to testing for the G×E when G and E are independent (i.e., ρ = 0, Supplementary Note). This hypothesis can be tested by the statistic $T_{diff}={\left(\hat{\alpha }-{\hat{\beta }}_{1}\right)}^{2}/\mathrm{var}\left(\hat{\alpha }-{\hat{\beta }}_{1}\right)$, where $\hat{\alpha }$, ${\hat{\beta }}_{1}$, and their corresponding standard errors are estimated from the GWAS and GWIS analyses, respectively. In fact, $T_{diff}$ and $T_{direct}$ are equivalent when GWAS and GWIS are performed in the same data. We verified this property using real data analysis in the GLI studies ^17^, from which the summary statistics of the marginal, main, and interaction effects are available and the marginal effect was obtained after adjusting for E (Fig S1). However, GWAS is often performed in a much larger sample than the GWIS because of data availability. The environmental exposure may have different distributions in cohorts for conducting GWAS and GWIS (i.e., different mean and variance). Furthermore, models (1) and (2) are likely to be performed by two different groups of investigators, which will bring variation across studies in trait definitions, trait measurement procedures, quality control procedures, and covariates. Moreover, the summary statistics are obtained through meta-analyses in both GWAS and GWIS analyses, which can bring additional variation and confounding factors, including population stratification and cryptic relatedness, leading to a potentially invalid comparison between the marginal and main effects. In fact, it has been reported that the confounding of population stratification is not sufficiently corrected in large GWAS ^26,27^. Therefore, directly using $T_{diff}$ to screen the genome can be biased even for testing the combined contribution of interaction and mediation.

**Fig. 1.**
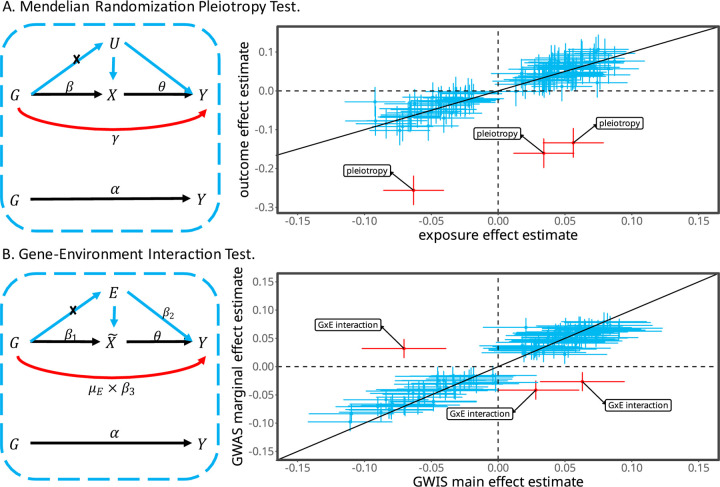
Illumination of Mendelian Randomization and G×E. **A**. Left panel: the path diagram of the MR, where U refers to all confounders. Genetic variants (*G*) contributing to outcome Y through mediation of exposure X are often selected as the valid genetic instrumental variables (black paths). Genetic variants contributing to Y through both black and red paths independently are horizontal pleiotropic variants. Right panel: the horizontal pleiotropic variants depart from the regression line and can be detected. **B**. Left panel: the G×E framework, with the goal of testing G×E. Instead of an explicit exposure, we create a pseudo exposure $\tilde{X}$, which can be viewed as the total contribution of genetic variants to trait Y. The genetic variants associated with the pseudo exposure $\tilde{X}$ but not through either the environment E or G×E are valid instrumental variables. The genetic variants interacting with E can be viewed the same as horizontally pleiotropic variants in the MR framework. Genetic variants associated with Y via mediation through *E* can contribute to both the pseudo exposure $\tilde{X}$ and Y, and thus have similar effects as G×E and cannot be distinguished from G×E. Thus, testing the combined effect of interaction and mediation is conceptually equivalent with testing the horizontally pleiotropic effect in the MR framework. Right panel: like the horizontal pleiotropic variants in the MR framework, G×E variants depart from the regression line and can be detected assuming no medication.

To overcome this bias, we note that the marginal effect estimate $\hat{\alpha }$ and the main effect estimate ${\hat{\beta }}_{1}$ have a linear relationship,

$$\hat{\alpha }=\theta {\hat{\beta }}_{1}+\frac{\rho \sigma_{E_{1}}}{\sigma_{G_{1}}}{\hat{\beta }}_{2}+\left(\mu_{E_{1}}+\frac{\rho \sigma_{E_{1}}}{\sigma_{G_{1}}}\right){\hat{\beta }}_{3},$$

where θ reflects the contribution of main effect to marginal effect, which converges to 1 when GWAS and GWIS are conducted using homogeneous measurements of phenotypes and environments (Online Methods). The genetic variants with no G×E and no mediation will fall on the regression line but the variants with G×E or mediation will depart from this line. This pattern will not be impacted by the systemic variation across studies. Therefore, we search the genetic variants that depart from this regression line to test the combined effect of G×E and mediation, providing θ can be correctly estimated. This idea is conceptually the same as the MR framework when we introduce a pseudo exposure $\tilde{X}$, representing a polygenic score of the trait (Fig 1). We then estimate the causal effect θ of the pseudo exposure $\tilde{X}$ on trait Y in the MR framework and the G×E effect or mediation through E is tested in the same way as testing for horizontally pleiotropic variants ^24^. In doing so, we first select a set of independent variants associated with trait Y and perform the inverse variance weighted analysis to estimate θ, denoted as $\hat{\theta }$. Second, we test the G×E or mediation of a genetic variant through E by the statistic $T_{MR\_GxE}=\frac{{\left(\hat{\alpha }-\hat{\theta }{\hat{\beta }}_{1}\right)}^{2}}{\mathrm{var}\left(\hat{\alpha }-\hat{\theta }{\hat{\beta }}_{1}\right)}∼\chi_{1}^{2}$. This test can be performed by the iterative Mendelian randomization and pleiotropy (IMRP) approach^24,28^. The statistic $T_{MR\_GxE}$ is an asymptotically unbiased test for testing the combined effect of G×E and mediation through E (Supplementary Note).

### Two-step procedure for Testing G×E

Note that $T_{MR\_GxE}$ likely tests for the combined effect of G×E and mediation unless G and E are independent (i.e., ρ=0). To test for G×E, we propose a two-step procedure by applying $T_{MR\_GxE}$ to screening the whole genome and then performing $T_{direct}$ on the variants surviving the $T_{MR\_GxE}$ screen. This two-step procedure can increase power at the screening step when there is interaction and mediation and increases power at the testing step by substantially reducing the multiple comparison burden. $T_{MR\_GxE}$ and $T_{direct}$ are not independent (Supplementary Note), and therefore, the variants detected by the two-step procedure could still reflect the contribution of mediation and G×E. To mitigate this problem, we can exclude the variants identified through GWAS of E, which could have large mediation effects.

### Type I error rate and power of $T_{\mathrm{MR\_GxE}}$ and the two-stage procedure

**Fig. 2.**
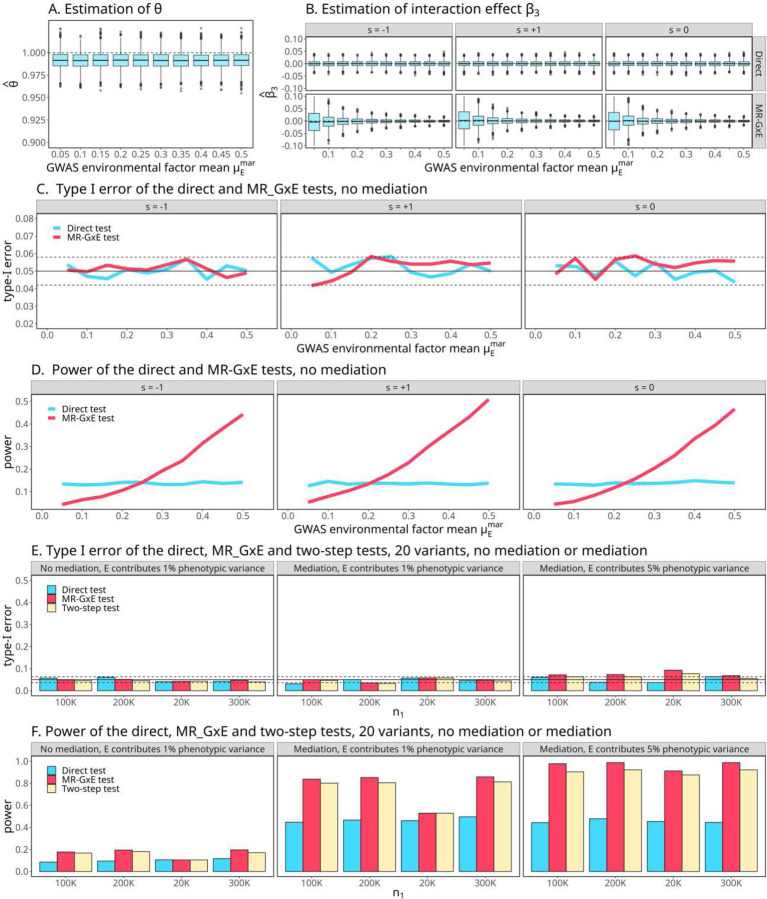
Simulation performance of 𝑻_𝑴𝑹__𝑮𝑿𝑬 and the two-step procedure. **A-D**: No medication was present. The simulation details were described in Online Methods. **A**. $E\left(\hat{\theta }\right)$ is close to 1 as expected. **B**. The direct estimate of $\beta_{3}$ in GWIS or by $\left(\hat{\alpha }-{\hat{\beta }}_{1}\hat{\theta }\right)/\mu_{e}$ through MR analysis are both unbiased. Here s=-1 refers to the scenario when the main effect and interaction effect have opposite effect directions; s=0 refers to no main effect; and s=1 refers to the scenario when the main effect and interaction effect have the same effect direction. **C**. Type I error rate comparison between $T_{MR\_GxE}$ and the direct test for different main and interaction effect directions. Both $T_{MR\_GxE}$ and the direct test maintain the type I error rate well. **D**. Power comparison between $T_{MR\_GxE}$ and the direct test for different main and interaction effect directions. **E-F**: 20 variants were tested when mediation was present or not. The simulation details were described in Online Methods. **E**. Type I error comparison for $T_{Dir}$, $T_{MR\_GxE}$ and two-step precedure. **F**. Power comparison for $T_{Direct}$, $T_{MR\_GxE}$ and two-step precedure.

We first performed a series of simulations to investigate the type-I error rate and power of $T_{MR\_GxE}$ in the absence of mediation (ρ=0). In simulations we observed that $E\left(\hat{\theta }\right)$ is close to 1 and the estimate $\hat{\theta }$ converges to 1 when sample size increases, which is expected by our theoretical prediction (Fig. 2A and Fig. S2). The interaction effect $\beta_{3}$ can be estimated directly by model (1) or by $\left(\hat{\alpha }-{\hat{\beta }}_{1}\hat{\theta }\right)/\mu_{E}$ when ρ=0. We observed that both ways are unbiased (Fig, 2B), although the standard error of $\left(\hat{\alpha }-{\hat{\beta }}_{1}\hat{\theta }\right)/\mu_{E}$ is affected by the environmental means in GWAS and GWIS. When no mediation is present, the type-I error rates for both $T_{MR\_GxE}$ and the direct test are well controlled (Fig. 2C and Fig. S2c)). The power of $T_{MR\_GxE}$ depends on multiple parameters, including $\mu_{E}$ and allele frequency in GWAS and GWIS and is less powerful than $T_{Direct}$ when the environmental mean in GWAS is lower (Fig. 2D and Fig. S2d). Additional simulations for the estimates of $\hat{\theta }$, interaction effect $\left(\hat{\alpha }-{\hat{\beta }}_{1}\hat{\theta }\right)/\mu_{E}$, type-I error rate and power are presented in Fig. S3-S5.

We next investigated the performance of $T_{Direct}$, $T_{MR\_GxE}$ and the two-step procedure when mediation is present and multiple variants are tested. We generated 20 independent variants with one variant having mediation, interaction or both. All three tests have well controlled type I error rates when mediation is absent (Fig. 2E and Fig. S6). When mediation is present, the type-I error rate was still well controlled, although inflation can be observed for the two-step procedure and $T_{MR\_GxE}$ when E contributes to 5% of the outcome variation and the samples between GWAS and GWIS are completely overlapped (Fig S6). This inflation was caused by mediation and quickly disappeared when the overlapping rate between GWAS and GWIS subjects was reduced. The statistical power of $T_{MR\_GxE}$ and the two-step procedure for testing G×E was much more improved than $T_{Direct}$ when mediation was present (Fig. 2F and Fig. S6).

### Identifying gene-smoking and gene-alcohol drinking interactions to serum lipids

We applied the two-step procedure to search for genetic variants interacting with cigarette smoking and alcohol drinking for serum lipids, using the summary statistics of high-density lipoprotein cholesterol (HDL-C), low-density lipoprotein cholesterol (LDL-C), and triglycerides (TG) from the GLGC (n=1.65M) and the CHARGE GLI (n=134K). To mitigate the effects of mediation through cigarette smoking or alcohol drinking, we excluded all loci with P-value <5×10^-7^ reported in the early GWAS of cigarette smoking status or alcohol drinking ^29^, which represent relatively large effect sizes of variants on cigarette smoking and alcohol drinking. We observed that $\hat{\theta }$ ranged from 0.92–1.33, 0.95–1.62, 0.83–1.25, 0.87–1.37, and 0.95–1.28 for European, African, Asian, Hispanic, and Cross-population data, respectively (Table S1). The departure of $\hat{\theta }$ from 1 suggests that the phenotype treatments, analysis protocols, and corrections for population structure were not identical between the GLGC and CHARGE GLI consortiums. For example, CHARGE GLI performed a natural logarithmic transformation to the lipid measurements, whereas GLGC further performed an inverse normal transformation. The number of principal components (PCs) for correcting populations was also different between GLGC and CHARGE GLI. Despite these discrepancies, we did not observe an inflation for $T_{MR\_GxE}$, with the genomic control λ values being close to 1 (range 0.93–1.05, Table S2).

**Fig. 3.**
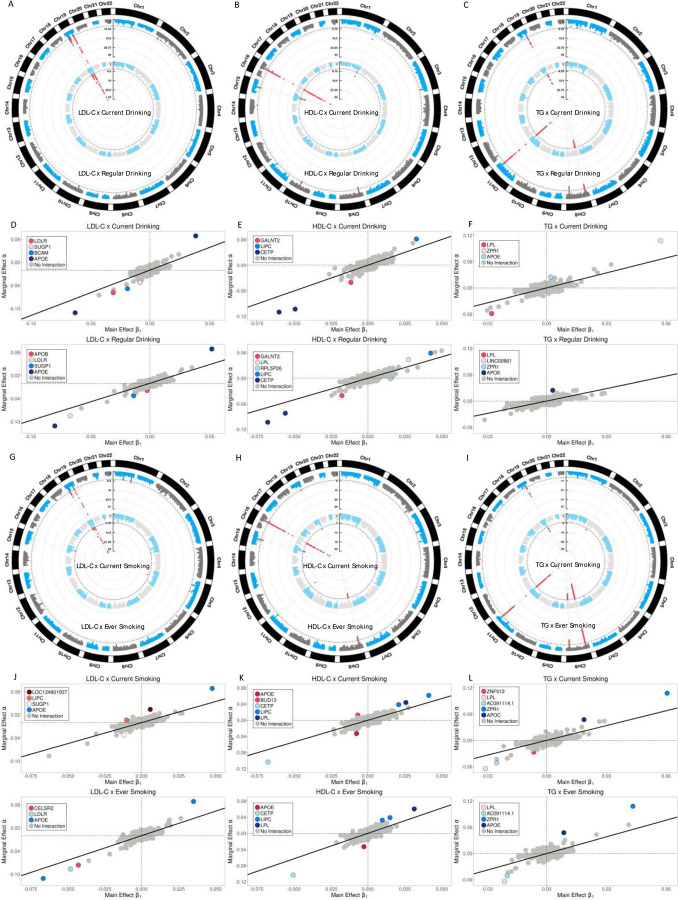
Manhattan plots for interactions, marginal and main effect size comparisons. **A-C**: The circle Manhattan plots of gene × alcohol drinking interactions for LDL-C, HDL-C, and TG. The genome wide significant loci are presented in red dots. **D-F**: The marginal and main effect size comparisons for LDL-C, HDL-C, and TG. The colored circles represent the genome-wide significant loci and gray circles represent the insignificant loci by $T_{MR\_GxE}$ test. **G-I**: The counterparts of gene × cigarette smoking for LDL-C, HDL-C, and TG, respectively.

Using $T_{MR\_GxE}$ to screen the genome, we observed 15 genome-wide significant loci consisting of 17 independent signals (P<5×10^-8^, *r*^2^ < 0.1), including 4 and 5 loci for LDL-C, 7 and 5 loci for HDL-C, and 5 and 6 loci for TG, interacting with cigarette smoking and alcohol drinking or mediating through them, respectively (Fig. 3A-C, G-I, Table S3). All but 3 loci have been reported to be associated with either cigarette smoking or alcohol drinking in the recent largest GWAS study with over 3 million samples ^30^, suggesting the contribution of both G×E and mediation. Since we already excluded the cigarette smoking and alcohol drinking variants identified from a relatively smaller study ^29^, these detected variants should represent modest mediation effects. Locus specific plots of all significantly associated loci are presented in Fig. S7, which suggest that multiple protein-coding genes are present in these loci. Strikingly, all the loci have previously been mapped to lipids traits except *RPL5P26* on chromosome 10. The G×E or mediation loci are clearly departing from most of the lipids-associated variants (Fig. 3D-F, J-L). The population-specific $T_{MR\_GxE}$ results are presented in Fig. S8, which are also consistent with the Cross-population results, although the main contribution comes from the European population.

By applying the two-stage procedure, we observed that 8 of the 17 independent signals are significant when using the direct test $T_{Direct}$ after correcting for the 17 tests and 4 environmental factors (Table 1, P<7.35×10^-4^). Tissue enrichment analyses using GWAS-based pathway analysis tools, MAGMA^31^ and FUMA ^32^, suggest that these loci are enriched in liver, hippocampus, small intestine, and stomach tissues (Fig. S9). Multiple loci were colocalized with expression quantitative trait loci (eQTLs) in the corresponding liver, lung, and blood tissues in the genotype-tissue expression database (GTEx) ^33^ (Fig. S10).

### Independent replication

We next attempted to replicate the evidence for these 8 independent signals in the UKBB. Although the UKBB data accounted for about one third of samples in the GLGC consortium, the direct test statistic $T_{Direct}$ calculated in the UKBB is independent of $T_{MR\_GxE}$, so are the 𝑇_𝐷𝑖*r*𝑒𝑐𝑡_ test statistics calculated in UKBB and CHARGE GLI, thus qualifying this as an independent replication (Supplementary Note). Six of the 8 signals were replicated in the UKBB after adjusting for 32 tests (P<1.56×10^-3^), 5 of which were genome wide significant by the $T_{Direct}$ test in combined CHARGE GLI and UK Biobank data (Table 1). All 8 independent signals had the same interaction direction in CHARGE GLI and UKBB except *LPL*, which is not significant in UKBB (Table S3). The *CETP* and *SMARCA4* loci were the only two loci with no reported mediation evidence through either cigarette smoking or alcohol drinking.

We next aimed to understand if the interaction evidence observed in this study had an alternative explanation ^34^ because of linkage disequilibrium (LD) with a variant which has causal effect on cigarette smoking or alcohol drinking. To examine this, we searched if there exists a variant(s) at each of the loci in Table 1 explaining the observed interaction evidence in the UKBB. However, we did not observe such variants (Fig S11), suggesting that the interaction evidence presented in Table 1 is genuine. In total, we identified 5 loci consisting of 6 independent signals that have evidence of interaction with either cigarette smoking or alcohol drinking.

### G×E Interaction and Mediation to SNP Heritability

**Fig. 4.**
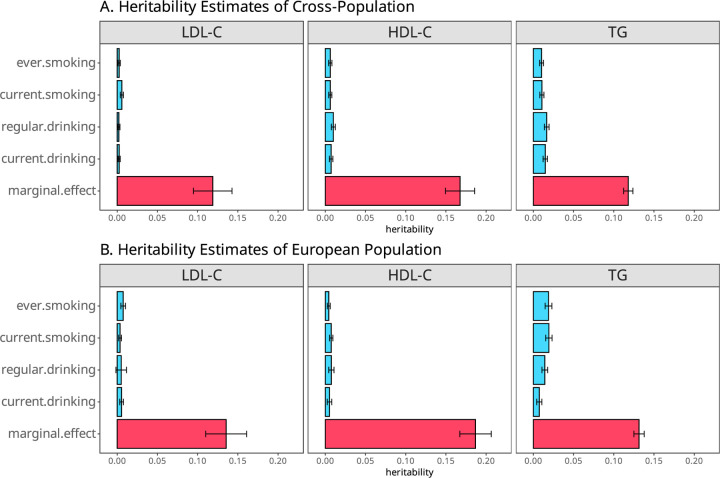
The estimated heritability of Cross-Population (A) and European population (B) using the LDSC regression. Marginal effect heritability refers to the heritability estimates through the marginal effect $\hat{\alpha }$ (red bars), and interaction effect heritability refers to the heritability estimated through $\hat{\alpha }-\hat{\theta }{\hat{\beta }}_{1}$ (blue bars). Only cross-population and European population are presented. The rest population specific heritability is presented in Table S4.

Since $\hat{\alpha }-{\hat{\beta }}_{1}\hat{\theta }$ refers to the combined interaction and mediation contribution to the marginal effect, we can use $\hat{\alpha }-{\hat{\beta }}_{1}\hat{\theta }$ to estimate the heritability contributed by the interaction and mediation through the LD score (LDSC) regression ^35^. Note that this heritability is a lower bound of the phenotype variance contributed by the G×E and mediation through E and is a part of the heritability estimated through the marginal effect, which is often referred to as the SNP-heritability in GWAS. We observed significant interaction and mediation heritability (P<1.93×10^-3^) with cigarette smoking and alcohol consumption to lipids traits, ranging from 1.76% (LDL-C, regular drinking) to 14.05% (TG, regular drinking) of SNP heritability estimated by the marginal effects (Fig. 4, Table S4).

### G×E Interaction and Mediation to heterogeneity of genetic effect sizes across populations

**Fig. 5.**
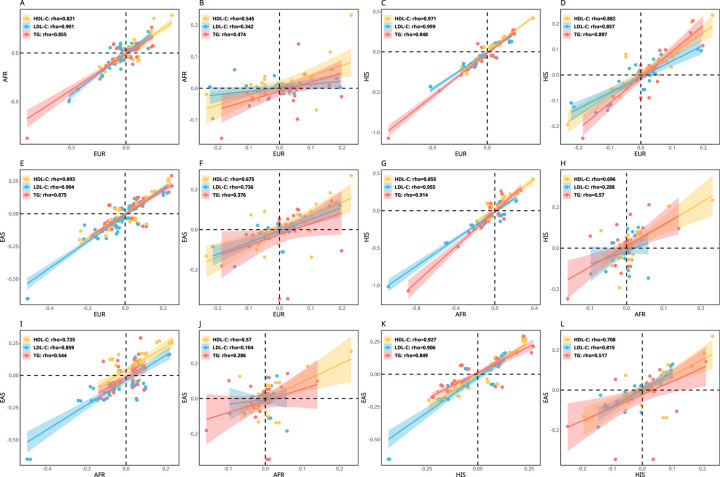
Cross population comparison of the LDL-C, HDL-C and TG marginal effect sizes of the variants reported in Graham et al ^3^ and the independent variants in Table S3 where their G×E interactions or mediations were observed in this study. The correlations in the first and the third columns represent the variants reported in Graham et al while the correlations in the second and fourth columns represent the variants in Table S3. Clearly the variants without G×E interactions or mediations have substentially larger cross population correlations than the variants with, suggesting that G×E interactions or mediations contribute the marginal effect size hetergeniety across populations. (European (EUR), African (AFR), Hispanics (HIS), Eastern Asian (EAS)).

As noted in equation (3), the marginal effect estimate of a genetic variant in GWAS consists of the G×E and mediation contribution when the G×E or mediation occurs. Because of the environment heterogeneity across populations, we expected that the marginal effect sizes of the variants will be less correlated across populations for the variants with G×E interaction or mediation than without. We calculated the marginal effect size correlations between European, African, Hispanic, and Eastern Asian populations for these variants reported in Graham et al ^3^ after excluding the variants in Table S3 where their G×E interactions or mediations were observed in this study. Similarly, we calculated the marginal effect size correlations for the variants in Table S3 using the effect sizes reported in Graham et al ^3^. We compared the correlations and observed a median of 24.4% drop of the cross-population correlation coefficient (Fig 5), strongly suggesting that G×E interactions or mediations contribute to the marginal effect size hetergeniety across populations.

## Discussion

To the best of our knowledge, this study is the first to utilize marginal effects from GWAS to search for G×E. We conceptually demonstrated the deep connection between detecting G×E and MR for causal inference. Although $T_{MR\_GxE}$ tests for the combined effect of G×EG×E and mediation, the two-step procedure of $T_{MR\_GxE}$ followed by $T_{Direct}$ in fact tests for G×E, and its statistical power is much improved when mediation is present and type I error rate is reasonably controlled. Detecting G×E using direct tests can be biased by unmeasured confounders due to omitting covariates in the regression models ^36^, but the new two-stage procedure is robust because $T_{MR\_GxE}$ is not affected by confounders such as population structure.

Our study demonstrated that the current heritability estimates based on marginal effects also include significant contributions from G×E and mediation through the corresponding environment factors (Fig. 4 and Table S4). We excluded cigarette smoking- or alcohol drinking-associated variants identified from a large cigarette smoking and alcohol consumption GWAS of 1.2 million individuals ^29^ in our analysis, which mitigates the potential mediation contribution in the $T_{MR\_GxE}$ analysis. However, among the 15 loci identified by $T_{MR\_GxE}$, only three were not reported in the much larger recent cigarette smoking and alcohol consumption GWAS of 3.4 million individuals, suggesting mediation through cigarette smoking and/or alcohol consumption is still present but with modest effects. Among the six G×E variants identified, 4 are associated with either cigarette smoking or alcohol drinking, suggesting that the G×E variants are also likely to be mediated through E and the mediation improves power to detect G×E. Furthermore, we demonstrated that the current SNP heritability estimates based on marginal effects also include significant contributions from G×E and mediation through the corresponding environment factors. We therefore suggest that the current SNP heritability estimates based on the marginal genetic effects be called marginal or broad-sense SNP heritability, to differentiate it from narrow-sense heritability ^37^ that is defined by additive genetic actions without the inclusion of G×E or mediation contributions. We believe this differentiation is important for correctly interpreting the current heritability estimates and understanding the genetic architecture of complex traits.

The 5 replicated loci (6 independent signals) interacting with cigarette smoking or alcohol consumption contain genes that are enriched in liver tissue, possibly reflecting the effect of alcohol drinking on aspartate amino transferase, alanine aminotransferase and γ-glutamyl transferase activities via the actions of numerous ingredients that alter the activities of enzymes found in the liver ^38^. Among them, the interaction between alcohol consumption and cholesteryl ester transfer protein (*CETP*) has been reported for HDL-C and coronary artery disease ^39–41^. The interaction between alcohol consumption and *APOE* on LDL-C has also been reported in a Mediterranean Spanish population ^42^, while the interactions between *APOA5* and cigarette smoking and alcohol drinking status associated with elevated TG and reduced HDL-C were observed in the Chinese and Korean populations ^43,44^. However, our study is the only well powered study demonstrating significant evidence at the genome wide level and the interaction loci are replicable. *SMARCA4* was reported to be associated with LDL-C in the lipids GWAS in Africans ^45^ but not in the recent largest lipids GWAS which is predominantly European ancestry ^3^. Overall, the marginal effect sizes of the variants are less correlated across populations for the variants with G×E interaction or mediation than without (Fig 5), empirically verifying that G×E and mediation contribute to marginal effect differences across different populations ^46^. We expect that including G×E interactions should improve polygenetic risk score prediction across populations.

It is well known that causal effect estimates in the MR framework can be biased when any of the three IV assumptions are violated. However, the MR-based G×E approach is less likely to be biased for these reasons: 1) the effect sizes of IVs on the pseudo exposure are all highly significant in GWAS, which represent strong IVs. 2) It is less likely to have a confounding effect between a trait and its pseudo exposure, i.e. a polygenic score. 3) The iterative Mendelian randomization and pleiotropy test is a powerful method to detect pleiotropy when the two IV conditions are satisfied ^24^ and, in particular, it is expected that most of the IVs do not interacted with the environmental factor *E*.

In summary, our novel G×E approach is powerful and able to detect genetic loci interacting with environments that account for significant phenotypic variability. Our findings indicate that the contribution of G×E in lipids is not ignorable. Our study only focuses on the interactions of genes with cigarette smoking and alcohol consumption in lipids. The cumulative interaction contribution with many environmental factors can even be greater. Detecting individual genetic loci with environmental interactions facilitates a better understanding of the genetic architecture of complex traits and can improve phenotype prediction.

## Online Methods

### Summary statistics Data

The marginal summary statistics of high-density lipoprotein cholesterol (HDL-C), low-density lipoprotein cholesterol (LDL-C), and triglycerides (TG) from the Global Lipids Genetics Consortium study (GLGC, n=1.65M) ^3^ were downloaded at http://csg.sph.umich.edu/willer/public/glgc-lipids2021. GLGC consists of GWAS results from 1.65M subjects representing five genetic ancestry groups: European (N = 1.32M); African or admixed African (N = 99K); East Asian (N = 146K); Hispanic (N = 48K); and South Asian (N = 41K). We did not perform South Asian specific analysis because there was no corresponding GWIS in the CHARGE consortium. The GWIS summary statistics from CHARGE GLI working group in this study are available via dbGaP (accession number phs000930).

### QCs for performing ${\text{T}}_{\text{MR\_GxE}}$ analysis.

To perform MR analysis, we merged the GWAS summary statistics for HDL-C, LDL-C, and TG from the GLGC with the corresponding GWIS summary statistics from the CHARGE GLI consortium. We flipped the effect size if the corresponding reference allele did not match. We dropped the genetic variant if its two alleles were either {A, T} or {C, G}. We also excluded any variants with minor allele frequency (MAF) difference larger than 0.15 between GLGC and CHARGE GLI studies. If multiple variants fell on the same chromosome position, we required the matched variants with MAF difference less than 0.01. We further excluded any variants with the effective sample size in GLGC trans-ethics or European less than 100K and the other populations (African, Hispanic, East Asian) less than 30K. To reduce the effect by mediations through the smoking and alcohol drinking, we excluded all loci with P-value < 5E-7 identified by the GWAS of smoking status or alcohol drinking^29^.

### ${\text{T}}_{\text{MR\_GxE}}$ analysis

To perform $T_{MR\_GxE}$, we applied the Mendelian randomization (MR) software IMRP ^24^ to estimate the causal effect by considering the main effect sizes from the GWIS of the CHARGE gene-lifestyle consortium as the exposure effects, and the marginal effects from the GLGC as the outcome effects, respectively. To identify instrumental variables, we first selected all the variants with the P-value < 5E-8 after GC-correction in the GLGC, and then pruned them using the window size 500 KB and *r*^2^ value 0.1 by the Plink software ^47^. We standardized the effect sizes as in ^28^. IMRP requires the input of the correlation coefficient to account for the effect of sample overlapping between GWAS and GWIS cohorts and this correlation was calculated based on the variants with P-value > 0.05) across the genome^48^. After estimating the causal effect, we performed $T_{MR\_GxE}$, which is equivalent to the pleiotropy test in the IMRP, to all the genetic variants across the genome.

### Independent locus definition.

Independent loci were defined as the regions within 1Mb of the most significant variants by the $T_{MR\_GxE}$ test. Independent signals were defined as the variants in a locus with *r*^2^ < 0.1. The 1000G data was used as the reference genetic data for LD calculation.

### Choosing independent variants for replication in UK Biobank

By applying $T_{MR\_GxE}$, we observed that 15 genome-wide significant loci consisting of 17 independent signals (P-value < 5E-8), including 4 and 5 loci for LDL-C, 7 and 5 loci for HDL-C, and 5 and 6 loci for TG, interacted with alcohol drinking and cigarette smoking, respectively (Table S3). At a locus with significant $T_{MR\_GxE}$ test for a lipid trait (LDL-C, HDL-C or TG) and environment (smoking or alcohol drinking), we searched the variant with the smallest P-value of the direct test $T_{Direct}$ among the significant variants by the $T_{MR\_GxE}$. The variants with $T_{Direct}$ P-value < 7.35E-4, which correct for the 17 tests and 4 environmental factors, were considered as significant for G×E interaction in the two-step procedure. We observed 8 independent signals in 6 loci among all 17 independent signals surviving the threshold P-value = 7.35E-4. These variants were further tested for the replication of the interaction effects in UK Biobank using $T_{Direct}$ test.

### LD Score Regression

We applied the LD score regression^35^ to estimate heritability contributed by G×E interaction and mediation through the environment factor E. To account for potential heterogeneity, we first estimated chromosome specific heritability and then summed across chromosomes. We used the R package bigsnpr ^49^ to estimate LD scores with the 1000G Phase 3 reference data and then estimated the chromosome-specific heritability with default settings. We observed that some of the chromosome specific heritability estimates were negative. We only summed the non-negative chromosome specific heritability estimates.

### Functional Mapping and Annotation.

We performed overall enrichment tests using the residual ${\hat{\alpha }}_{j}-{\hat{\beta }}_{j}\hat{\theta }$ as the effect size and $se\left({\hat{\alpha }}_{j}-{\hat{\beta }}_{j}\hat{\theta }\right)$ as the corresponding standard error. We used MAGMA ^31^ (Multi-marker Analysis of GenoMic Annotation) and DEPICT ^50^ (Data-driven Expression Prioritized Integration for Complex Traits) to identify tissues and cells that are highly expressed at genes within the G×E loci. We also used DEPICT to test for enrichment in gene sets associated with gene ontology (GO) ontologies, mouse knockout phenotypes and protein-protein interaction networks. In addition, we reported significant enrichments with a false discovery rate 0.05. Analysis was done using the online platform FUMA GWAS.

### Colocalization

We performed colocalization analysis by using the software ezQTL^51^. We chose the public genotype-tissue expression (GTEx) v7 with eQTL ^33^ as the QTL data and chose the public European reference panels for calculating the LD data. We performed colocalization analysis between GWIS and QTL results within a locus using eCAVIAR (eQTL and GWAS Causal Variant Identification in Associated Regions) ^52^, where the Colocalization Posterior Probability (CLPP) was used to describe the significance level of colocalization. We only recorded colocalization with CLPP > 0.01, as suggested by the authors of eCAVIAR.

### UK Biobank individual level data for replication

The UK Biobank (UKBB) ^53^ individual-level data used for replications were available through Application ID: 81097. Quality Controls Participants in the UKBB were genotyped using a custom Affymetrix UK Biobank Axiom array ^54^. Genotypes were imputed by the UKBB using the Haplotype Reference Consortium reference panel ^55^ with imputation *r*^2^ value greater than 0.3. Related individuals with pairwise kinship coefficient greater than 0.0884 (suggested by UKBB) were removed from analysis, resulting in N = 445,424 individuals of European, African, and East Asian ancestries. For a pair of related individuals, one was randomly excluded. The principal components were calculated by UKBB with genotype data within each ancestry to account for population structure. These data were independent of GLI cohorts and consisted of European, African, and Asian individuals (race determined using UKBB field ID 21000–0.0). Linear regression model (1) in the main text was performed. Covariates included age at assessment (21003–0.0), age^2^, sex (31–0.0), the first 10 PCs (22009–0.1 to 22009–0.10), the environmental exposure, a genetic variant and their interaction. Environmental exposures included ever/never smoking status (20116–0.0), current/non-current smoking status (20116–0.0), and alcohol intake frequency (1558–0.0).

Analogous to the G×E analysis in ^17^, HDL-C (30760–0.0) and TG (30870–0.0) measurements were natural log transformed and LDL-C measurements (30780–0.0) were converted from mmol/L to mg/dl then multiplied by a factor of 0.7 if there was a history of lipid-lowering medication (6177–0.0) present. LDL-C measurements therefore did not consider medication if there were missing values for medication history. This introduced missing values in LDL-C for 248,419 individuals.

### Theoretical properties of $T_{\mathrm{MR\_GxE}}$

In MR analysis, the instrumental variables are independent and are genome wide significant variants selected from GWAS. Let ${\hat{\beta }}_{1,j}$, ${\hat{\beta }}_{2,j}$, ${\hat{\beta }}_{3,j}$ and ${\hat{\alpha }}_{j}$, j=1,…,m, be the corresponding effect size estimates in GWIS (model (1) and GWAS (model 2)) for the m instrument variables.

The causal effect θ of the inverse variance weighted (IVW) is estimated by

$$\hat{\theta }=\arg\min\left\{\frac{1}{m}\sum_{j=1}^{m}\frac{{\left({\hat{\alpha }}_{j}-{\hat{\beta }}_{1,j}\theta \right)}^{2}}{\text{var}\left({\hat{\alpha }}_{j}\right)}\right\}.$$

It is much simpler to work on $\hat{\theta }$ by standardizing the IVs and this procedure does not change the conclusion. Thus, we let $\sigma_{G,j}^{2}=1$, j=1, …, m, in both GWAS and GWIS data. Further, we let the phenotype residue variance $\sigma^{2}=\;1$. By equation (S15) in Supplementary Note, we have $\text{var}\left({\hat{\alpha }}_{j}\right)=n_{1}^{-1}$, j=1,…,m, and $\hat{\theta }=\frac{\sum_{j=1}^{m}{\hat{\alpha }}_{j}{\hat{\beta }}_{1,j}}{\sum_{j=1}^{m}{\hat{\beta }}_{1,j}^{2}}$.

Since only the variants without either G×E interaction or mediation are valid in the MR analysis, we assume ρ=0 (no mediation) and $\beta_{3,\text{j}}=0$ (no interaction). We have

$${\hat{\alpha }}_{j}={\hat{\beta }}_{1,\text{j}}+\mu_{E1}{\hat{\beta }}_{3,\text{j}}$$

By applying Slutsky’s theorem, and let $\beta_{3,\text{j}}=0$, we have:

$$E\left(\hat{\theta }\right)=\frac{\frac{1}{m}\sum_{j=1}^{m}E\left({\hat{\alpha }}_{j}{\hat{\beta }}_{1,j}\right)}{\frac{1}{m}\sum_{j=1}^{m}E\left({\hat{\beta }}_{1,j}^{2}\right)}=\frac{\frac{1}{m}\sum_{j=1}^{m}\beta_{1,j}^{2}+\frac{n_{0}}{n_{1}n_{2}}\left(1+\frac{\mu_{E0}^{2}}{\sigma_{E0}^{2}}\right)}{\frac{1}{m}\sum_{j=1}^{m}\beta_{1,j}^{2}+\frac{1}{n_{2}}\left(1+\frac{\mu_{E2}^{2}}{\sigma_{E2}^{2}}\right)}.$$

Because $\sigma_{G,j}^{2}=1$, $\frac{1}{m}\sum_{j=1}^{m}\beta_{1,j}^{2}$ is the average phenotypic variance accounted by an IV. Define $\sigma_{\beta }^{2}=\frac{1}{m}\sum_{j=1}^{m}\beta_{1,j}^{2}$, we have:

$$E\left(\hat{\theta }\right)=\frac{\sigma_{\beta }^{2}+\frac{n_{0}}{n_{1}n_{2}}\left(1+\frac{\mu_{E0}^{2}}{\sigma_{E0}^{2}}\right)}{\sigma_{\beta }^{2}+\frac{1}{n_{2}}\left(1+\frac{\mu_{E2}^{2}}{\sigma_{E2}^{2}}\right)},$$

which converges to 1 when $n_{1}$ and $n_{2}\to \infty$. However, when $\sigma_{\beta }^{2}$ is small (weak instrument in MR analysis), the converge of $E\left(\hat{\theta }\right)$ to 1 is slow. We also note that $E\left(\hat{\theta }\right)\leq 1$.

We propose $T_{MR\_GxE}$,

$$T_{MR\_GxE}=\frac{{\left(\hat{\alpha }-\hat{\theta }{\hat{\beta }}_{1}\right)}^{2}}{\text{var}\left(\hat{\alpha }-\hat{\theta }{\hat{\beta }}_{1}\right)},$$

for testing the combined effect of G×E interaction and mediation: $\frac{\rho \sigma_{E}}{\sigma_{G}}\beta_{2}+\left(\mu_{E}+\frac{\rho \sigma_{E}}{\sigma_{G}}\right)\beta_{3}=0$. $T_{MR\_GxE}$_*E*_ can be performed by the IMRP method in MR analysis^24^.

Our procedure to test for the G×E interaction is: 1) We apply $T_{MR\_GxE}$ to search variants with joint effect of mediation and interaction effect; 2) we apply $T_{Direct}$ for the variants detected by $T_{MR\_GxE}$. To reduce the effect by the mediation, we can exclude the variants associated with E.

### Simulation settings without medication contribution (Fig 2A-D and Fig. S2-S5).

For the 𝑖 th individual, we generated 𝑚 = 102 independent variants for j=1,…,m by $G_{ij}^{*}∼\text{Binom}(2,p_{j})$, where $p_{j}∼\text{unifom (0.05,0.5)}$. We standardized genotypes by $G_{ij}=\frac{G_{ij}^{*}}{2p_{j}\left(1-p_{j}\right)}$. For the environment factor in the GWAS model, we generated $E_{i1}∼𝒩(\mu_{E1},1)$. For the environment factor in the GWIS model, we generated $E_{i2}∼𝒩(\mu_{E2},1)$. For the samples overlapped between the GWAS and GWIS, we generated their environment values through $𝒩(\mu_{E2},1)$. We varied the values of $\mu_{E1}$, $\mu_{E2}$ and the proportion of overlapped samples.

The main effect size of the 𝑗th variant was generate by $\beta_{1j}∼𝒩(0,\sigma_{\beta }^{2})$, where $\sigma_{\beta }^{2}$ is the trait variance accounted for by the IVs. For the first variant, we added its interaction effect with E. The phenotype $Y_{i}$ by generated by

$$Y_{i}=\sum_{j=1}^{m}G_{ij}\beta_{1j}+0.1E_{i}+0.05(G_{i1}*E_{i1})\;+\;\epsilon_{i},$$

where $\epsilon_{i}∼𝒩(0,\sigma^{2})$. The causal effect θ was estimated using the last 100 variants as the IVs. The power and type I error rate for $T_{Direct}$ and $T_{MR\_GxE}$ were calculated based on the first and second variants, respectively.

### Simulation settings without medication contribution (Fig 2E-F and Fig S6).

We generated 20 independent variants by $G_{j}$ Binom(2,0.3) and standardized them but without mean correction. We simulated environment E according to mediation present or not present. If no mediation, E was generated from 𝒩(1,1). If there was mediation, E~0.05G+𝒩(2,0.9975), or G contributes 0.25% the variation of E. The phenotype was generated according to the following models:

1. No mediation and no interaction: $Y\sim 0.1G+\gamma E+N\left(0,10\right)$, where E~𝒩(1,1)
2. Mediation but no interaction: $Y\sim 0.1G+\gamma E+N\left(0,10\right)$, where E~0.05G+𝒩(1,0.9975)
3. Mediation and interaction: $Y\sim 0.1G+\gamma E+0.1G*E+𝒩\left(0,10\right)$, where E~0.05G+𝒩(1,0.9975)

We let γ take values of 1 and $\sqrt{5}$. We also simulated data with environment mean 0.5 (Fig. S6). We first simulated $n_{2}$ = 20,000 subjects for GWIS cohort (or main effect estimation). The sample size for marginal effect estimation varied from $n_{1}$ = 20,000 to 300,000, with the 20,000 subjects in GWIS cohort always included. For the non-overlapped subjects, we let the environment mean to be 1.5 times of the environment mean in GWIS cohort. The type I error and power for $T_{Direct}$ and $T_{MR\_GxE}$ were calculated by correcting for 20 tests using the Bonferroni correction. For the two-step procedure, we first applied $T_{MR\_GxE}$ and Bonferroni correction. The variants surviving after $T_{MR\_GxE}$ were further tested by $T_{Direct}$ and Bonferroni correction was further applied.

## Supplementary Material

Supplement 1

## Figures and Tables

**Table 1.** Interaction loci screened by $T_{\mathrm{MR\_GxE}}$ and followed by the direct test $T_{\mathrm{Direct}}$ in GLI (two-step test) and replicated by the direct test $T_{\mathrm{Direct}}$ in UK Biobank.

Mapping Gene	CHR: BP	Lead SNP	Environmental Factor	Lipid traits	MR_GxE Test P-value	GLI Direct Test P-value	UKBB Direct Test P-value	GLI and UKBB Direct Test P-value
Signals identified by $T_{MR\_GxE}$ (P<5E-08), by $T_{Direct}$ (P<7.35E-04) and replicated by $T_{Direct}$ in UKBB (P<1.56E-3) or combined GLI and UKBB $T_{Direct}$ P<5E-8								
*BUD13* *	11:116637146	rs12294259	Regular Drinking	TG	**2.47E-18**	**3.61E-06** ^a^	**1.97E-04** ^a^	**2.14E-08**
	11:116657561	rs3741298	Current Smoking	TG	**2.80E-13**	**1.16E-10** ^a^	4.24E-01^a^	**6.99E-10**
*CETP*	16:57000696	rs8045855	Current Drinking	HDL-C	**6.12E-24**	**1.85E-07** ^a^	**4.97E-07** ^a^	**4.05E-12**
	16:57006829	rs289713	Regular Drinking	HDL-C	**5.01E-19**	**3.63E-07** ^a^	**3.16E-06** ^a^	**4.62E-11**
*BCAM* *	19:45392254	rs6857	Regular Drinking	LDL-C	**4.02E-12**	**1.28E-06** ^a^	**2.95E-04** ^a^	**8.57E-09**
NECTIN2* *TOMM40 APOE APCO1*	19:45422946	rs4420638	Regular Drinking	LDL-C	**6.55E-36**	**4.41E-05** ^a^	**1.95E-06** ^a^	**2.08E-09**
*LPL* *	8:19830170	rs1569209	Current Smoking	TG	**4.77E-10**	**1.01E-13** ^b^	3.49E-02^b^	**1.04E-13**
*SMARCA4*	19:11191677	rs10402112	RegDrink	LDL-C	**1.85E-15**	**5.75E-04** ^a^	**9.04E-04** ^a^	8.04E-06
Signals identified by $T_{MR\_GxE}$ (P<5E-08) and by $T_{Direct}$ (P<7.35E-04) but failed in UKBB replication								
*RPL5P26* *	10:71533084	rs11591480	Regular Drinking	HDL-C	**3.34E-08**	**1.11E-04** ^a^	5.23E-02^a^	8.69E-05
*ZPR1* **	11:116662579	rs651821	Ever Smoking	TG	**7.34E-17**	**3.44E-05** ^a^	6.87E-01^a^	1.73E-04

* The locus has been reported to be associated with cigarette smoking

** The locus has been reported to be associated with both cigarette smoking and alcohol drinking

a The interaction effect direction is the same in GLI and UKBB. Detailed effect sizes and standard errors are presented in Table S3.

b The interaction effect direction is opposite in GLI and UKBB. Detailed effect sizes and standard errors are presented in Table S3.
