# Supplementary material for: A new Approach to Identify Gene-Environment Interactions and Reveal New Biological Insight in Complex traits
Source: Res Sq. 2023 Oct 5:rs.3.rs-3338723. Preprint. [Version 1] doi: 10.21203/rs.3.rs-3338723/v1 (PMC10602131; doi:10.21203/rs.3.rs-3338723/v1)
Supplement: Supplement 1 [file NIHPPRS3338723V1-supplement-1.pdf]

Supplementary Files

This is a list of supplementary files associated with this preprint. Click to download.

- SupplementaryTablesV18.xlsx
- SupplementaryMaterial.docx
